# The positive outlook study- a randomised controlled trial evaluating the effectiveness of an online self-management program targeting psychosocial issues for men living with HIV: a study protocol

**DOI:** 10.1186/1471-2458-14-106

**Published:** 2014-02-04

**Authors:** Tanya Millard, Julian Elliott, Sean Slavin, Karalyn McDonald, Sally Rowell, Sonya Girdler

**Affiliations:** 1 Central Clinical School, Department of Infectious Diseases, Monash University, Level 5, Alfred Centre, 99 Commercial Road, Melbourne 3004, Australia; 2 Infectious Diseases Unit, Alfred Hospital, Level 2 Burnet Tower, 85 Commercial Road, Melbourne 3004, Australia; 3 Burnet Institute, 85 Commercial Rd, Melbourne 3004, Australia; 4 Centre for Social Research in Health, University of New South Wales, Sydney 2052, Australia; 5 Australian Research Centre in Sex, Health and Society, La Trobe University, Melbourne, Australia; 6 Western Australian AIDS Council, PO Box 1510, West Perth 6872, Australia; 7 School of Occupational Therapy and Social Work, Centre for Research into Disability and Society, Curtin Health Innovation Research Institute, Curtin University, GPO Box U1987, Perth 6845, Australia

**Keywords:** HIV, Self-Management, Online Intervention, Quality of Life

## Abstract

**Background:**

The emergence of HIV as a chronic condition means that people living with HIV are required to take more responsibility for the self-management of their condition, including making physical, emotional and social adjustments. This paper describes the design and evaluation of Positive Outlook, an online program aiming to enhance the self-management skills of gay men living with HIV.

**Methods/design:**

This study is designed as a randomised controlled trial in which men living with HIV in Australia will be assigned to either an intervention group or usual care control group. The intervention group will participate in the online group program ‘Positive Outlook’. The program is based on self-efficacy theory and uses a self-management approach to enhance skills, confidence and abilities to manage the psychosocial issues associated with HIV in daily life. Participants will access the program for a minimum of 90 minutes per week over seven weeks. Primary outcomes are domain specific self-efficacy, HIV related quality of life, and outcomes of health education. Secondary outcomes include: depression, anxiety and stress; general health and quality of life; adjustment to HIV; and social support. Data collection will take place at baseline, completion of the intervention (or eight weeks post randomisation) and at 12 week follow-up.

**Discussion:**

Results of the Positive Outlook study will provide information regarding the effectiveness of online group programs improving health related outcomes for men living with HIV.

**Trial registration:**

ACTRN12612000642886.

## Background

Medical advances in the management of HIV have improved health outcomes for people living with HIV (PLHIV) and significantly reduced the morbidity and mortality associated with the disease. Consequently, the care of HIV positive individuals has developed into a chronic disease management model [1]. As with other chronic illnesses, PLHIV are required to cope with a wide range of changes to physical and psychological health, social relationships and medication regimes. In addition, the relative invisibility of HIV within many countries means that the condition is highly stigmatized, presenting numerous issues surrounding disclosure, intimate relationships and service access [2-4]. As there is presently no cure, HIV/AIDS continues to be a chronic condition affecting an increasing number of people around the world.

Service delivery for HIV is varied, focusing on case management models, medical models, and more recently, self-management models. PLHIV are required to attend three to six monthly medical appointments to monitor levels of HIV in the blood, immune status, potential medication side effects, receive preventive health interventions and obtain medication prescriptions. In addition to these medical appointments, PLHIV may access community based services and supports. However the majority of these services are located in the inner suburbs of large cities, restricting access for people in remote, rural or suburban locations and those with limited access to transportation [5]. Research into men’s health-related behaviors indicates that men are typically reluctant to seek information and support [5-8]. For men living with HIV, stigma is an additional and prominent barrier to their willingness to access HIV services and supports [9]. Consequently, many men living with HIV in Australia receive little HIV specific support other than that provided by their General Practitioner and/or HIV clinician.

The Positive Outlook study aims to develop a self-management intervention for men living with HIV and evaluate this intervention using a randomised controlled trial (RCT). Although many studies have employed RCT’s to evaluate the effectiveness of self-management programs [10-14] and online interventions [14-16], there is a paucity of research evaluating the efficacy of online self-management programs targeted to meet the needs of PLHIV. This study will result in the development of a standardised online intervention for men with HIV which could potentially be used Australia wide by HIV/AIDS organisations and other service delivery organisations. This article describes the Positive Outlook intervention and research design.

### Objective of the study

The primary objective of this study is to evaluate the effectiveness of the Positive Outlook program in improving health outcomes as measured by the Health Education Impact Questionnaire (heiQ), domain specific self-efficacy as measured by the Positive Outlook Self-efficacy Scale and HIV specific quality of life as measured by the PROQOL-HIV compared with usual care only. Secondary outcome measures include depression, anxiety and stress, general health and quality of life, adjustment to HIV and social support.

## Methods/design

### Theoretical framework

The Positive Outlook intervention was informed by a thorough needs assessment and developed according to self-management [17] and self-efficacy theory [18].

#### Self-management

As HIV has become a chronic condition, a self-management model where PLHIV assume an active, informed role in their health care may be beneficial. Self-management, as described by Lorig and colleagues [17], involves three tasks: medical management, role management and emotional management, and encompasses six skills: problem solving, decision-making, resource utilization, the formation of a patient-provider partnership, action planning and self-tailoring. In order to be a successful self-manager, motivation, healthy behaviors and effective collaboration with health professionals is required [19].

Programs which educate and support patients to manage their chronic condition, including the physical, emotional and social aspects, have successfully improved health outcomes [20]. In self-management programs, the client is central in taking responsibility for the management of their condition [21]. The World Health Organization [22] includes self-management as best practice to improve clinical care and outcomes for individuals with chronic illnesses. Self-management has been widely used and demonstrated effectiveness in the care of patients with diabetes [23-26], arthritis [10,27,28], vision loss [11], chronic obstructive pulmonary disease [29,30] and heart disease [31-33].

Benefits to be obtained from participating in self-management interventions include reduced morbidity, improved health related behaviors and health outcomes, reductions in use of acute medical services and hospital visits, and improved quality of life [29]. Clearly, self-management interventions improve patient outcomes in many areas while simultaneously reducing the burden on the health care system [20,34,35].

#### Self-efficacy

Self-efficacy is defined as an individual’s confidence in their ability to perform a specific task or achieve a certain outcome. According to Bandura [36], the ability to achieve positive outcomes and prevent negative outcomes is a powerful incentive for increasing perceptions of control. Perceived self-efficacy influences the behaviors people choose to undertake and the effort they apply to achieving their desired goals [36].

Growing evidence suggests that educational and behavioral interventions, including self-management programs can enhance self-efficacy [11,31]. Self-efficacy plays a major role in health behavior change for individuals with chronic illness and is considered a strong predictor of health promoting behavior [36,37]. Self-efficacy has also been linked with improving positive perceptions of health, faster recovery from illness and reduced levels of depression [38].

A recent systematic review [39] which sought to appraise the literature on self-management interventions for people living with HIV, identified only six interventions meeting the reviews selection criteria. The interventions meeting the inclusion criteria for the review were varied, some focused solely on symptom management and others had a broader approach targeting a wider range of topics. The review found evidence to infer that self-management programs for PLHIV may result in short term improvements in physical, psychosocial and health knowledge and behavioral outcomes [39]. Despite some very promising results, due to limitations in the quality of the data presented in the articles, firm conclusions regarding the efficacy of self-management programs for PLHIV to improve health and behavioral outcomes remain elusive. None of the programs provided evidence regarding the effectiveness of self-management programs improving social support, quality of life and the management of HIV within intimate relationships. These are the areas the Positive Outlook Program aims to address in an online format.

### Online delivery of health interventions

In recent years, the Internet has become a frequent source of health information [40]. The Internet is accessible 24 hours a day and is relatively cheap to use. In 2010–2011 79% of households in Australia had home internet access and 83% of households had access to a computer [41]. Research has identified the value of the Internet in providing PLHIV information on treatments, health management and living with HIV [42-44]. The HIV Futures 6 Survey identified the internet as the most important source of information regarding living with HIV for PLHIV in Australia, followed by their general practitioner, HIV specialist and HIV positive friends [42].

Interactive health information provides numerous benefits to individuals with chronic illnesses. Kalichman and colleagues [45] found that benefits included the opportunity for people to access and collect information at their own pace, anonymously ask ‘uncomfortable’ questions, identify coping resources and seek support as well as providing the opportunity for users to learn about treatment developments and options and to develop a sense of control over their condition.

There is growing evidence of the effectiveness of online interventions for people living with chronic conditions. Wantland and colleagues [14] conducted a meta-analysis comparing the effectiveness of web-based versus non web-based interventions for individuals with a variety of chronic illnesses. Through effect size comparisons of the web-based verses non web based interventions for 17 studies, participants receiving online interventions demonstrated improvements in knowledge and behavior change outcomes, including time spent exercising, knowledge of nutritional status, knowledge of treatments, participation in health care and body shape perceptions [14]. In addition, web-based participants demonstrated slower health decline and 18-month weight loss maintenance than those who received non-web based interventions.

### Online self-management

Due to advances in technology and access to computers and the internet, self-management programs have recently begun to be delivered online, however, research evaluating the effectiveness of online self-management programs is inconclusive. Lorig and colleagues [13] conducted a prospective longitudinal study to evaluate the effectiveness of an online self-management program for 443 English residents living with a variety of chronic conditions. Participants demonstrated a reduction in symptoms, improved health behaviors, self-efficacy and satisfaction with health services [13]. Health care utilization was also reduced and these benefits were maintained for a period longer than a year. Significant improvements were found at six months for all variables except self-rated health, disability, stretching, hospitalization and nights in hospital [13].

Research has also demonstrated the effectiveness of disease-specific online interventions. A recent randomized trial evaluated the effectiveness of an internet-based asthma self-management program in comparison to usual care [46]. Participants in the online intervention, improved in asthma related quality of life, asthma control, lung function and the number of symptom free days, compared to the ‘usual care’ control group [46]. Successful use of the internet as a delivery option has also been demonstrated in a RCT trial for patients with arthritis or fibromyalgia [16]. Results from this study indicated that at one year follow up, the intervention group demonstrated significant improvements in four of six health status measures and self-efficacy, compared to participants in the ‘usual care’ control group [16].

Although self-management programs have demonstrated several advantages, numerous barriers exist for individuals wishing to access them including finance, time, work, rural location and stigma [35]. At present there are no online self-management programs which specifically target the needs of PLHIV. Online health interventions provide individuals with the opportunity to overcome many of these barriers as they can access the information at the time and place that is convenient [40,47,48].

### Development of the intervention: needs assessment

The PRECEDE Model guided the needs assessment which underpinned the Positive Outlook program. This model provides a structure for the systematic application of theories and concepts for planning and evaluating health education and promotion programs [49]. The acronym PRECEDE stands for Predisposing, Reinforcing and Enabling Constructs in Educational/Ecological Diagnosis and Evaluation [50]. The model is used to identify health problem characteristics including the causes of the problem and the impact of health-related behaviour and the environment [51].

Objectives of this needs assessment were to identify the population (men living with HIV in Australia), assess the quality of life of the population and to uncover the behavioural and environmental factors impacting quality of life and priority areas for the men. In conducting a thorough needs assessment, the PRECEDE model promotes the use of numerous and varied information sources [51]. Accordingly, a multi-faceted needs assessment was conducted which used both qualitative and quantitative methodologies in order to enable an in-depth understanding of the impact of HIV on the lives of men living with HIV in Australia.

The needs assessment consisted of four steps. First, the literature was reviewed which examined the epidemiology and impact of HIV amongst men. Second, a survey assessing quality of life (QOL) of men living with HIV was conducted and results were compared to Australian normative data. Focus groups were then conducted to obtain a more in-depth description of the impact of HIV on QOL and perceived problem areas in daily life. A focus group was also conducted with a multidisciplinary team of service providers working in the area of HIV to obtain their perspective of the impact of HIV and areas of need. Finally, a systematic literature review was conducted which examined the effectiveness of existing HIV specific self-management interventions and identified those programs and models which could be used to inform the intervention targeting the needs of this group.

The findings of the needs assessment indicated that psychosocial issues are prominent among this population. Priority areas were identified as the following: (1) Managing the emotional impact of HIV (2) Disclosing HIV status to family and friends (3) Maintaining social connectedness (4) Managing HIV within intimate relationships (5) Disclosure of HIV status to intimate partners. Findings from this needs assessment will be reported elsewhere.

### The positive outlook intervention

The Positive Outlook program was developed by the primary researcher (TM) in consultation with an intervention team. The intervention team brought together people with expertise in HIV and self-management of chronic conditions. They provided feedback and recommendations to develop and refine the intervention.

The program is based on Bandura’s self-efficacy theory [18] and utilizes a self-management approach to enhance participants’ skills, confidence and abilities to manage the psychosocial aspects of HIV in their daily lives. The intervention encourages participants to take responsibility for the self-management of their condition including managing physical, social and emotional aspects of health and making behavioral changes.

The program is delivered as a closed group with approximately 15 people per group. Over seven weeks, participants log onto the intervention for approximately 90 minutes per week. The program is accessible through a website (http://www.positiveoutlook.org.au) which is password protected and consists of a series of information modules (comprised of 15–20 web pages) to be completed by the participants each week. Each module provides interactive self-management information as determined by the objectives identified in the needs assessment (See Table 1). Participants are required to complete a number of goal setting and action planning activities and to use discussion boards to communicate with other group members about their action plans, experiences, problems, progress and goal attainment. The discussion board also provides the opportunity for social support, modeling and group persuasion to occur; vital components of self-management and self-efficacy theory [17,37]. The program also contains a “live chat” feature whereby once a week at a designated time, participants are encouraged to log on simultaneously to discuss the week’s content.

**Table 1:** Program overview

	**Topic**	**Chat topic**
**Week 1**	Part 1: Orientation	Introductions and program expectations; Health goals.
	Part 2: Maintaining a healthy lifestyle	
**Week 2**	HIV and You: Emotional well being and social connectedness	Stress management and depression
**Week 3**	Talking About HIV: Disclosure of positive status to friends and family	Disclosure
**Week 4**	HIV, You and Others: HIV and intimate relationships; safe sex basics	Relationships
**Week 5**	HIV, You and Others: Disclosure to partners	Disclosure to intimate partners
**Week 6**	HIV, You and Others: Managing risk	Risks within intimate relationships
**Week 7**	Wrapping up: Review	Medication’s; Open discussion; Program review

Each group is facilitated by two peer support officers recruited from community organizations supporting PLHIV in Australia. Each facilitator receives a training session and an instruction manual describing their responsibilities. Facilitators use pseudonyms and do not have access to participants’ personal information. The facilitators are responsible for modeling desirable participation; facilitating and encouraging group discussion; moderating content on discussion boards; providing additional information; sending participants reminders where required via personal messages tab and providing feedback regarding participants goal setting and action planning activities. The facilitators also monitor and facilitate the weekly “live chat.” There is no predetermined timeframe for the facilitators to spend on the program, however, they are asked to commit a minimum of 30 to 45 minutes per day and up to two hours when facilitating the “live chat”. Participants are able to communicate directly and privately with the facilitators if required through a ‘private messages’ option on the website. Participants also receive weekly reminders of the program delivered via email and or SMS from an external facilitator (the primary researcher).

### Pilot study

A pilot study to test the feasibility of the Positive Outlook program and to provide both formative and process evaluation was conducted in June 2012. Twelve participants were recruited from the target population for this pilot study through the Western Australian AIDS Council. Ten participants completed baseline assessments and were enrolled to participate in the seven week Positive Outlook program. One participant withdrew in week five due to personal issues. Data was collected from the remaining nine participants immediately following the intervention. Six of the nine participants were also interviewed about their experience of using the online program. Minor adjustments were made to the content and delivery of the intervention based on the results of the pilot study.

A second pilot study was launched on the 22^nd^ October, 2012. This pilot group was intended to be the first of the RCT, however it was decided that a few additional changes were required to improve the program prior to trial. Participants completed baseline data and were randomised into the intervention (n = 19) or the control group (n = 17). Two participants withdrew in week three. Data was collected from 21 participants at the conclusion of the intervention. The findings of the pilot studies will be reported elsewhere. Participation on the discussion boards was extremely limited for the intervention group. Engagement with the Action Plans was also limited. After discussions with the facilitators of the program, interviews with the participants themselves and discussion with the intervention team, we decided to include a “live chat” into future versions of the program. We believed that the program was too flexible and the delay in responses was acting as a barrier for participation for many of the participants. By facilitating a live chat, participants will have more accountability for participation and will potentially get more out of instant responses and the “virtual” conversation whilst still maintaining their anonymity.

### Identification and recruitment of participants

Potential participants will contact the researcher directly in response to advertisements on Facebook, community organisation websites, advertisements in gay press, flyers and posters located in AIDS councils and other services for PLHIV, and GP offices and referral from health professionals. Potential participants will be directed to the Positive Outlook website (http://www.positiveoutlook.org.au) where they can register their interest by providing their name and email address on a secure server. Within one day, participants will be invited to join the study by the primary researcher (TM) and sent the electronic information and consent forms. Contamination of research participants is unlikely given the web-based nature of the intervention and use of individual login names and passwords. The primary researcher will ensure there is no double up of email addresses and postal addresses (where provided). Participants will also be recruited using multiple organisations, further reducing the chance of contamination of research participants.

### Inclusion and exclusion criteria

Inclusion criteria for participating in this study is as follows:

• HIV positive men over 18 years living in Australia

• Self identifies as gay, homosexual or MSM (men who have sex with men)

• Ability to read and write in English

• Self reported access to a computer and the internet for at least 90 minutes per week for seven weeks

• Adequate computer skills to enable participation.

### Randomisation

Participants who indicate consent will be allocated a unique participant identification number. Once sufficient participants are recruited for the first round of randomisation (n = 30), participants will be sent electronic baseline questionnaires and a registration form and be randomised to either the intervention or control condition. Participants will be allocated into either the control group or the intervention group using a computer generated list of random numbers [52]. A spreadsheet containing the participant’s unique identification numbers will be constructed. An associate researcher will allocate participants to the intervention or control group using the list of computer-generated numbers on the spreadsheet. The primary researcher (TM) is blinded to randomisation. Once randomisation has been completed, the primary researcher will email each participant advising them of their group allocation.

### Sample size calculations

A power calculation was undertaken based on findings from a study by Gifford et al. [53], which used self-efficacy for controlling symptoms as the primary outcome measure. This study employed a symptom self-efficacy scale developed specifically for measuring the impact of self-management interventions for chronic diseases, and closely reflects the self-efficacy measure developed for use in evaluating the Positive Outlook program. Following an RCT aimed at evaluating the effectiveness of a self-management in men with symptomatic HIV or AIDS, Gifford et al. reported that self-efficacy for controlling HIV symptoms increased in the intervention group, and decreased in the control group (+4 versus -7; p = 0.02) [53]. Based on these findings, a sample size of 52 in each group was found to be adequate to identify a mean difference in score of 11 (assuming a standard deviation of 19.8), with 80% power and using a α = 0.05. The RCT would then require a total sample size of N = 104. If there appear to be any differences in baseline characteristics between groups, a regression model may be used to adjust for these. The general ‘rule of thumb’ is that a sample size of approximately 120 would be adequate to identify covariates which exhibit a moderate effect size power = 80%). Therefore the RCT will aim to recruit at least N = 120, requiring four rounds of randomisation of 30 participants. The research team believes this is a feasible number to achieve.

### Data collection

#### Quantitative data

**Figure 1:**
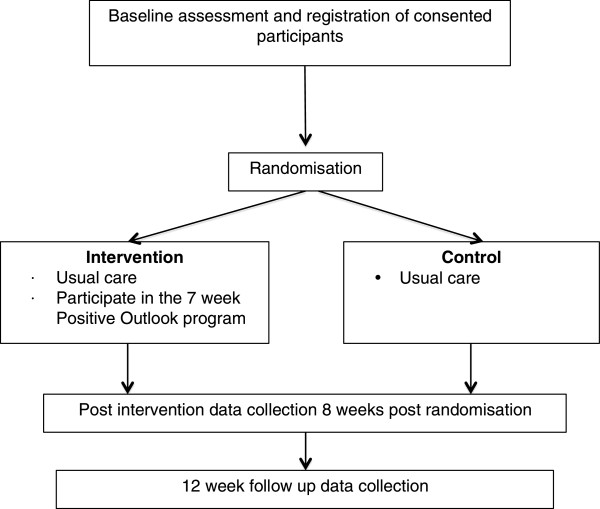
Flow chart of Positive Outlook Study intervention process.

Data will be collected online (via Survey Monkey) with the exception of the SF-12^®^ which the primary researcher (TM) will deliver via post or phone. Electronic questionnaires will be emailed to participants at three time points: baseline (Timepoint 1); immediately after the completion of the intervention or eight weeks post randomisation (Timepoint 2); and 12 weeks following the intervention (Timepoint 3). Demographic information will be collected at time one only and will include age, year of diagnosis, co existing conditions, relationship status, living situation and annual income. It is anticipated that data collection at each timepoint will take no longer than 45 minutes to complete. Participants will be given two weeks to submit completed follow up questionnaires. They will receive email, SMS and phone reminders as required. For a detailed flow chart on data collection, please see Figure 1.

#### Primary outcome measures

##### 

**Patient Reported Outcomes Quality of Life-HIV (PROQOL-HIV)** The PROQOL-HIV was developed to measure the health related quality of life of PLHIV in the current HAART era [54]. The PROQOL-HIV demonstrates good convergent and discriminant validity and reliability (8% scaling success; Cronbach alphas 0.77-0.89) [55].

##### 

**Outcomes of health education: Health Education Impact Questionniare (heiQ)** The Health Education Impact Questionnaire (heiQ) will be used to evaluate the effectiveness of the program. The heiQ evaluates patient education and self-management programs for chronic illness [56]. It has demonstrated high construct validity ranging from 0.70 to 0.83 for each of the dimensions [57].

##### 

**Domain specific self-efficacy: Positive Outlook Self-efficacy Scale** The Positive Outlook Self-efficacy Scale was specifically designed for this study to address a significant gap in the ability of existing standardised questionnaires to evaluate the outcomes targeted by this intervention. The authors’ wanted to use a singe questionnaire that assessed participants’ confidence in their knowledge, skills and abilities pertaining to the physical, social and emotional aspects of life with HIV. Confidence in ability to disclose HIV and to use risk reduction strategies within intimate relationships in particular, are not assessed in any current standardised assessments. This scale is comprised of 19 questions which are broken down into five individual dimensions including knowledge, communication, relationships, social participation and emotions. The primary researcher (TM) composed the scale based on existing chronic condition self-efficacy scales [58]. Questions were developed targeting the specific aims of the Positive Outlook Program. The contributing authors then further refined the questionnaire. Minor adjustments were made to the scale following piloting by the research team and two HIV positive peer support officers. As this is a new scale, validity and reliability of data using this scale cannot be assured. Results obtained from this scale will therefore be interpreted and reported with caution.

#### Secondary outcome measures

##### 

**Health related quality of life: Short-Form Health Survey Volume 2 (SF-12v2^®^)** The SF-12^®^ consists of eight subscales including: physical functioning, role limitations due to physical health problems, bodily pain, social functioning, general mental health, role limitations due to emotional problems, vitality and general health perceptions [59]. The psychometric properties have been tested and proved sound amongst a variety of general and disease specific populations (reliability coefficient median 0.76; relative validity median = 0.97) [59]. It is also considered a valid measure for use with PLHIV [60].

##### 

**Depression Anxiety and Stress: Depression Anxiety and Stress Scale (DASS)** The DASS is a 21-item standardised self-report measure consisting of three scales measuring depression, anxiety and stress. In a large clinical sample (N = 437) the DASS demonstrated high levels of internal consistency (alpha = 0.96, 0.89 and 0.93) for depression, anxiety and stress respectively [61].

##### 

**Social Support: Duke Social Support Index (DSSI)** The 11-item DSSI was derived from the longer, 35-item DSSI and consists of a subjective evaluation of the adequacy of support received as well as a more objective evaluation of type and number of social interactions [62]. Goodger and colleagues [62] conducted a study evaluating the reliability and validity of the scale in a sample of 565 community dwelling people aged 70 years and over. They reported that the scale demonstrated good internal consistency (alpha. = 0.77), and test-retest reliability (n = 117) from 0.07 to 0.81.

##### 

**Domain specific adjustment: Mental Adjustment to HIV Scale (MAH)** The MAH scale is based on the Mental Adjustment to Cancer (MAC) scale [63] which has demonstrated validity and reliability in a range of cancer populations [64]. Ross and colleagues [65] reported on the use of the MAH in a population of 107 Australian men with HIV. They reported that the five subscales of the MAH demonstrated adequate Cronbach’s alpha reliabilities between 0.55 and 0.8 [65].

#### Statistical analysis

Descriptive statistics will be used to characterise the sample at baseline. Unpaired t-tests and chi-square tests will detect any differences between the control and intervention groups at baseline. Analysis of covariance (ANCOVA) will be used to evaluate changes in the dependent variables at post-test and follow up, controlling for differences between the groups at baseline. Data analysis will use an intention-to-treat approach. All analyses will be conducted using SPSS for Windows.

#### Qualitative data

Case studies of 12 men from the intervention groups will be conducted to explore in depth, the experiences of participating in the program. The sample will be composed of three men from each intervention group. Sampling of participants for the case studies will be purposive, with the aim to obtain a range of successful and unsuccessful outcomes as measured by the assessment battery. Data collection will involve semi-structured interviews conducted via telephone completed within one week of program completion. Semi-structured interviews will also be conducted with each facilitator involved in the delivery of the Positive Outlook program. Interviews will be digitally recorded and transcribed verbatim. Data will be de-identified and analyzed using the constant comparative method of qualitative data analysis [66]. The primary researcher will also keep field notes for the duration of the RCT.

#### Process evaluation

Process evaluation will consist of a mixed methods approach using both quantitative and qualitative data as recommended for process evaluation of RCT’s [67]. Website data (number of logins; duration of logins; pages visited; modules completed; discussion board posts and attendance at live chats per participant) will be used to evaluate participation and adherence to the program. At the conclusion of the program, participants will be asked to complete an evaluation survey regarding their experiences using the Positive Outlook Program. Questions will include outlining the strengths and weaknesses of the program; suggestions for improvement; satisfaction with the website; level of participation in the program and overall satisfaction with the program. Participant and facilitator interviews as described above will also contribute to process evaluation.

### Trial organisation and management

The proposed study will be conducted in accordance with the ethics guidelines as explained in the National Statement on Ethical Conduct in Human Research [68]. Ethics approval has been granted by the Human Research Ethics Committee at Monash University, Edith Cowan University, The Alfred Hospital, ACON and the Victorian AIDS Council. The trial has been registered with the Australia and New Zealand Clinical Trial Registry #00362515.

### Trial status

No data cleaning or analysis of the RCT has been executed prior to submission of this manuscript.

## Discussion

We describe here the design of the Positive Outlook study, a randomised controlled trial evaluating the effectiveness of an online self-management program for gay, homosexual and other MSM living with HIV. The intervention is based on self-efficacy theory and uses a self-management approach to enhance participants’ confidence, skills and abilities to manage many of the psychosocial issues associated with living with HIV.

This study is significant for several reasons. Meeting the health care needs of individuals with HIV is an important public health issue. The Australian Government is strongly committed to the self-management of chronic conditions, listing self-management support as a key component of routine care [69]. Self-management requires changes to the physical, emotional and social aspects of ones life [17]. Due to significant advances in the effectiveness of ART, the physical effects of HIV are relatively well managed by medications. In contrast, more investment is required to address the psychological and social issues that continue to impact the lives of people living with HIV. For example, issues surrounding stigma, negotiating intimate relationships and disclosure are, in many ways, unique to the experience of living with HIV and thus require targeted attention in order to be adequately addressed and managed [20,39,70].

Existing self-management interventions for PLHIV have addressed adherence to medications, medication side-effects and symptom management, physical exercise, nutrition, stress, anxiety and self-efficacy [71-77]. Findings from a recent systematic review of self-management education programs for PLHIV indicated that, while such programs may result in short-term improvements in physical, psychosocial, health knowledge and behavioral outcomes, further research needs to be devoted towards establishing the effectiveness of programs improving social support, the management of HIV within intimate relationships and quality of life [39].

To our knowledge, in Australia, the only available self-management program for PLHIV is the Stanford program (Positive Self Management Program) [53], offered through the Bobby Goldsmith foundation in Sydney. This program is more general in its approach and an evaluation of the program noted its focus on improving participants self efficacy, which can result in group roles being forced upon participants and little attention being given to participants’ own issues because they do not fit within with the syllabus [70]. The program is not presently offered online, limiting access for people who may encounter barriers to participation, including stigma, time, geographical location and transport.

Other programs available in Australia utilizing self-management elements, such as life coaching and peer support, are currently available through various community organizations supporting PLHIV. However, the majority of these groups are targeted towards, and accessed by, those newly diagnosed. Given the prominence of psychosocial issues for PLHIV, particularly regarding disclosure and negotiating intimate relationships, it is clearly important that these issues be addressed to enhance the quality of life of PLHIV in Australia.

The model of care for PLHIV has long been based on one of empowerment, encouraging patients to take an active and informed role in their health care. Gay men have been widely recognized as advocates for their health and community in their response to HIV. In dealing with the HIV epidemic, gay men have developed a strong sense of agency and lead the way in forming partnerships with health care providers, advocating for one’s self and adopting a ‘peer support’ model of care [78]. Peer support and education programs have been widely used for PLHIV [78]. Benefits of peer support include the low cost of implementation and the general accessibility and availability as opposed to care provided by health care professionals. The shared experiences, circumstances and common characteristics amongst peers further facilitates their appeal and effectiveness [78].

The present intervention specifically targets the five priority areas identified in the needs assessment: (1) Managing the emotional impact of HIV (2) Disclosing HIV status to family and friends (3) Maintaining social connectedness (4) Managing HIV within intimate relationships (5) Disclosure of HIV status to intimate partners. The two pilot studies assisted the intervention team to further develop and refine the program in order to maximize participants’ engagement and satisfaction with the program. The main changes included the addition of the ‘Live Chat’ component and a reduced focus on the Action Planning Activities.

The most recent National HIV Strategy (2010–2013) called for a reduction in the “transmission of and morbidity and mortality caused by HIV and to minimize the personal and social impact of HIV” [79]. In recognition of their commitment to achieving the overall goal and priority actions identified in this strategy, community organizations across Australia are well positioned to adopt and promote an online self-management style program in order to improve the QOL of PLHIV.

## Conclusion

This study is the first of its kind to apply the theoretical framework of self-efficacy and self-management theory to an online group intervention for PLHIV in Australia. The online format of the Positive Outlook Program will potentially increase access to marginalized populations living with HIV throughout Australia. In addition to improving quality of life and health outcomes of gay, homosexual and MSM living with HIV, this study will also provide valuable in sight into the feasibility of online delivery of group interventions for PLHIV.

## Competing interests

The authors declare that they have no competing interests.

## Authors’ contribution

TM, JE, KM, SS, SR, SG contributed to the design of the study. TM drafted the manuscript. TM, JE, KM, SS, SR, SG reviewed the manuscript. The manuscript has been read and approved by all authors.

## Pre-publication history

The pre-publication history for this paper can be accessed here:

http://www.biomedcentral.com/1471-2458/14/106/prepub
